# Prostate MRI based on PI-RADS version 2: how we review and report

**DOI:** 10.1186/s40644-016-0068-2

**Published:** 2016-04-11

**Authors:** Philipp Steiger, Harriet C. Thoeny

**Affiliations:** Department of Radiology, Neuroradiology, and Nuclear Medicine, Inselspital, Bern University Hospital, University of Bern, Freiburgstrasse 10, CH-3010 Bern, Switzerland; Department of Diagnostic, Interventional and Pediatric Radiology, University Hospital of Bern, Inselspital, Freiburgstrasse 10, CH-3010 Bern, Switzerland

**Keywords:** Prostate cancer, Multiparametric magnetic resonance imaging (mpMRI), Prostate Imaging Reporting and Data System version 2 (PI-RADS™ v2)

## Abstract

Prostate imaging and interpretation is based on prostate imaging reporting and data system version 2 (PI-RADS™ v2) providing clinical guidelines for multiparametric magnetic resonance imaging (mpMRI) of the prostate. PI-RADS™ v2 aims to promote global standardisation, to diminish variation in the acquisition, interpretation and reporting of prostate mpMRI examinations and to improve detection, localisation, and risk stratification in patients with suspected cancer in treatment naïve prostate glands. It does not address detection of recurrence, progression during active surveillance and evaluation of other parts of the body.

PI-RADS™ v2 improves and standardises communication between radiologists and urologists to detect or exclude the presence of significant prostate cancer with a high likelihood. Findings on mpMRI are assessed on a 5-point category scale based on the probability that a combination of findings on T2-weighted (T2w) sequences, diffusion-weighted MRI (DWI) and dynamic contrast-enhanced MRI (DCE-MRI) correlates with the presence of a clinically significant prostate cancer at a particular location. PI-RADS assessment categories range from 1 to 5 with 5 being most likely to represent clinically significant prostate cancer. The dominant sequence to detect prostate cancer in the peripheral zone is DWI, whereas for tumour detection in the transition zone T2w is the most important sequence. DCE-MRI has been attributed a minor role and only qualitative assessment with presence or absence of focal enhancement is suggested. Up to four suspicious lesions of category 3, 4 and 5 are assigned on a sector map and the index lesion should be identified.

## Introduction to clinical context and proposed utility of modality

Prostate cancer is the second most common cancer in men worldwide [1]. Two thirds of prostate cancer cases are diagnosed in the more developed regions of the world. The incidence varies over a broad range among different countries probably as a result of a difference in the number of men screened. Mortality of prostate cancer is difficult to measure due to the mortality of concomitant cardiovascular diseases and other cancers which share similar risk factors and therefore act as confounders. The prevalence of latent prostate cancer at autopsy is high and occult carcinomas are common [1]. According to two big screening studies “Prostate cancer screening in the randomized Prostate, Lung, Colorectal, and Ovarian Cancer Screening Trial” (PLCO) [2] and “Screening and prostate cancer mortality: results of the European Randomised Study of Screening for Prostate Cancer” (ERSPC) [3] overdiagnosis is common, therefore screening for prostate cancer is controversial. Prostate cancer screening is based on digital rectal examination (DRE) and prostate-specific antigen (PSA) level testing in the blood serum. PSA is highly sensitive but not specific for prostate cancer. Benign pathologies such as benign prostatic hyperplasia can raise PSA levels and normal PSA levels can not exclude prostate cancer [4]. Prostate biopsies are assessed histologically by the Gleason score, a prognostic factor of prostate cancer, which provides information on tumour aggressiveness [5]. Prostate cancer is divided into clinically insignificant and significant prostate cancer, depending on its likelihood to affect a patient’s lifetime. There is no universal definition for clinical significant prostate cancer, but the most frequent applied definition is the following: Pathology/histology with Gleason score ≥ 7 (including 3 + 4 with prominent but not predominant Gleason 4 component), and/or volume ≥ 0.5 cc, and/or extra prostatic extension (EPE) [6]. Depending on the Gleason score and clinical assessment of the patient’s disease, treatment options for prostate cancer are active surveillance, surgery and radiotherapy [7].

MRI became the method of choice for detection and staging of prostate cancer [8]. Adapted from breast imaging a “Prostate Imaging Reporting and Data System” (PI-RADS) was published by the European Society of Urogenital Radiology (ESUR): PI-RADS™ version 1 [9] (PI-RADS™ v1). This first guideline paper was based on a summary score for each lesion assessed in different sequences of mpMRI, consisting of T2w, DWI and DCE-MRI and spectroscopy facultatively. These guidelines have been updated recently by a steering committee including the American College of Radiology (ACR), ESUR and the AdMeTech Foundation to the PI-RADS™ v2 [10, 11]. In this version spectroscopy was omitted and DCE-MRI was attributed a minor role. In contrast to version 1 each lesion is attributed a single score based on findings of mpMRI. The objectives of these guidelines were to promote global standardisation of prostate imaging, to improve detection, localisation, characterisation, risk stratification of prostate cancer in treatment naïve prostate as well as to improve communication with referring urologists. The latest PI-RADS version assesses the likelihood (probability) of clinically significant prostate cancer on a 5-point scale for each lesion as follows:

- PI-RADS 1 – Very low (clinically significant cancer is highly unlikely to be present)
- PI-RADS 2 – Low (clinically significant cancer is unlikely to be present)
- PI-RADS 3 – Intermediate (the presence of clinically significant cancer is equivocal)
- PI-RADS 4 – High (clinically significant cancer is likely to be present)
- PI-RADS 5 – Very high (clinically significant cancer is highly likely to be present)

**Fig. 1:**
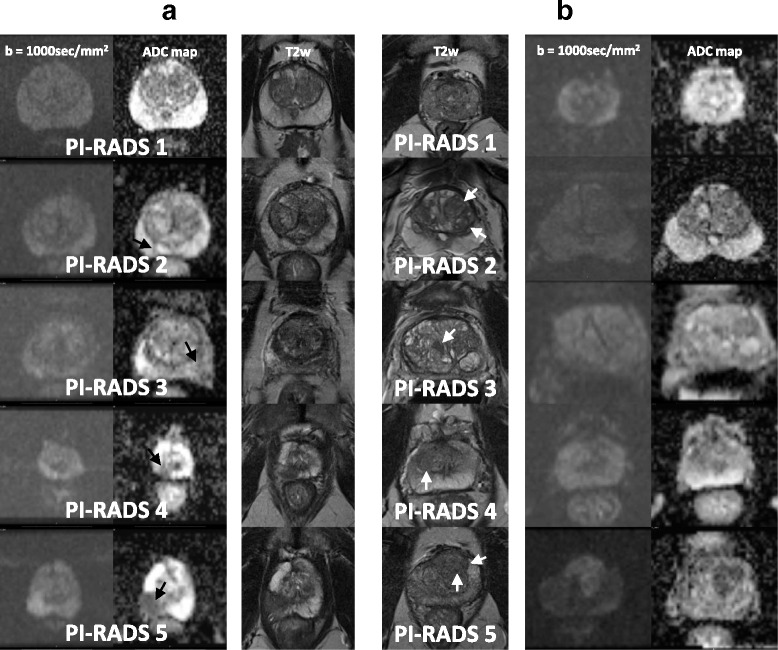
PI-RADS™ v2 assessment categories: Fig. 1**a** depicts typical findings in the peripheral zone and Fig. 1**b** depicts typical findings in the transition zone. **a**. Peripheral zone shows PI-RADS assessment categories with DWI as dominant sequence. 1. No abnormality (i.e. normal) on the high b-value DW image and on the corresponding ADC map as well as on the T2w image. 2. Isointense signal of the peripheral zone on the high b-value DW image with an indistinct linear hypointense lesion on the ADC map (arrow) with corresponding T2w hypointense signal. 3. Isointense/mildly hyperintense signal of the peripheral zone on the high b-value DW image and with a focal mildly/moderately hypointense indistinct lesion on the ADC map (arrow). T2w image shows heterogeneous signal intensity of the peripheral zone. 4. Focal markedly hyperintense lesion on high b-value DW image with corresponding markedly hypointense signal intensity on the ADC map (arrow). Lesion size is < 1.5 cm on axial images. T2w image shows a circumscribed homogenous hypointense lesion. 5. Same as 4, but lesion size is ≥ 1.5 cm in greatest dimension (arrow). Definite extraprostatic extension/invasive behaviour (not shown) would also qualify for this category. **b** Transition zone shows PI-RADS assessment categories with T2w as dominant sequence: 1. Homogeneous intermediate signal intensity (normal) on T2w image and no abnormality on high b-value DW image and ADC map. 2. Circumscribed (arrows) hypointense or heterogeneous encapsulated nodule(s) (BPH). High b-value DW image shows normal signal intensity and indistinct hypointense signal on ADC map. 3. Lesion with heterogeneous signal intensity with obscured margins (arrow). High b-value DW image of the lesion is mildly hyperintense and moderately hypointense on the ADC map. This category includes lesions that do not qualify as 2, 4, or 5. 4. Lenticular (arrow) or non- circumscribed, homogeneous, moderately T2w hypointense lesion, < 1.5 cm in greatest dimension. High b-value DW image of the lesion shows markedly hyperintense signal and markedly hypointense signal on the ADC map. 5. Same as 4, but lesion size is ≥ 1.5 cm in greatest dimension (arrows). Definite extraprostatic extension/invasive behaviour (not shown) would also qualify for this category

For corresponding examples of findings see Fig. 1.

Up to four findings of category 3, 4 or 5 on mpMRI are mentioned and the index (dominant) lesion with the highest PI-RADS assessment category is determined. A final PI-RADS score is reported for each lesion.

Indications for prostate MRI include patients with clinical suspicion of prostate cancer (elevated PSA > 4.0 ng/mL and/or suspicious DRE) before biopsy for tumour detection or exclusion, patients with known prostate cancer for staging purposes (scheduled for treatment with curative intent or active surveillance) as well as patients with a negative biopsy but continuous clinical suspicion of prostate cancer.

MRI serves as an instrument for detection and/or staging of prostate cancer at the same time. In case of a suspicious lesion detected on MRI, targeted MRI-transrectal ultrasound (TRUS) fusion guided transperineal or transrectal biopsy is performed after meticulous demonstration of the findings to the referring urologist, as previously described [12].

Detection of recurrence after radiotherapy or radical prostatectomy, progression during active surveillance or for detection of metastases outside the pelvis are not considered in PI-RADS™ v2.

MRI not only offers an excellent resolution of the prostatic gland, but also evaluates locoregional extension, pelvic lymph nodes involvement and bone metastases in the pelvis. Ultrasound alone has too poor spatial resolution to suffice as an adequate imaging method. C11- and F18- choline positron emission tomography (PET) are other imaging methods used for detection of recurrence or metastatic disease in prostate cancer, but not for detection of the primary tumour or local staging. New tracers, such as Ga68 prostate-specific membrane antigen may play a role in prostate cancer imaging in the future as well as a combination of PET/MRI [13].

### MRI protocol

Patients with contraindications for MRI have to be excluded. If possible patients should evacuate the rectum just prior to the MRI exam. For reduction of bowel motion artefacts an antispasmolytic agent is administered. MRI of the prostate is performed according to the PI-RADS™ v2 guidelines on a 3 T scanner without an endorectal coil. The prostate including the seminal vesicles are imaged as follows with a reduced field of view:

Axial high-resolution T2w sequence with the following imaging parameters: Repetition time (TR) = 3710 msec, echo time (TE) = 113 msec, slice thickness = 3 mm, pixel size = 0.4 × 0.4 mm, field of view (FoV) = 220 mm and acquisition time (TA) = 4 min 29 s.

Axial DWI sequence with b-values of 0, 500, 1000, 1500 and 2000 s/mm^2^ and with the following imaging parameters: TR = 4700 msec, TE = 93 msec, slice thickness = 3.5 mm, pixel size = 3.1 × 3.1 mm, FoV = 160 mm and TA = 6 min 37 s.

Axial DCE-MRI sequence with a standard, preferable macrocyclic, gadolinium based contrast agent administered with a dose of 0.1mml/kg. Injection rate is 2.5 cc/s starting with continuous image data acquisition with a temporal resolution of 3.4 s and acquisition time up to 283.5 s. Imaging parameters are as follows: TR = 4.22 msec, TE = 1.35 msec, slice thickness = 3.5 mm, pixel size = 1.4 × 1.5 mm, FoV = 220 mm and TA = 4 min 46 s.

A precontrast T1w sequence is needed to exclude haemorrhage. We perform it from the aortic bifurcation to the pubic symphysis with the following imaging parameters: TR = 3.92 ms and TE = 1.24 msec, slice thickness = 2 mm, pixel size = 1.3 × 1.8 mm, FoV = 400 mm and TA = 21 s. In addition a high resolution T2w sequence should be performed in the coronal and sagittal plane. We prefer a 3D coronal T2w sequence of the pelvis with an isotropic voxel of 1 mm to allow reconstruction in the axial and the sagittal plane. Imaging parameters are as follows: TR = 1500 msec, TE = 122 msec, slice thickness = 1 mm, pixel size = 1.0 × 1.0 mm and FoV = 380 mm and TA = 7 min 2 s. This sequence includes the aortic bifurcation and the pubic symphysis and facilitates reconstruction in any plane especially for lymph node staging in combination with an additional DWI sequence of the entire pelvis with b-values of 0, 500, 1000 s/mm^2^ and with the following imaging parameters: TR = 10300 msec, TE = 50 msec, slice thickness = 4 mm, pixel size = 3.1 × 3.1 mm, FoV = 420 mm and TA = 5 min 03 s.

All sequences are acquired without breath-hold. For ADC map calculation b-values (0 - 1000 s/mm^2^) were used.

Finally, though not part of the PI-RADS™ v2 guidelines, the entire pelvis is scanned after contrast medium administration with an isotropic T1w fat saturated sequence with a voxel size of 1.2 mm. The reason for this additional sequence is to better evaluate other extraprostatic findings.

### Display of images

**Fig. 2:**
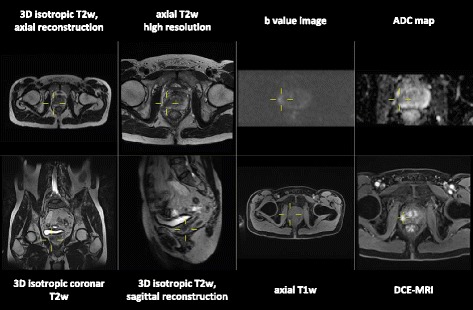
Standard layout of our prostate image display in a one screen setting. Double clicking in one of the images allows to display this image in full screen mode

Ideally two 21.0 inch or one 30.0 inch standard radiology monitor for image display with a resolution of at least 3 megapixels and a contrast ratio of at least 750:1 should be available. Additional monitors for reporting and other activities e.g. PACS steering, Internet, reference databases are recommended. Depending on the software, which is used, morphologic and functional sequences should be displayed at the same time. For a first overview axial T2w images are recommended. On the left side we display the morphologic T2w images. On the right side DWI is displayed, i.e. b-value images and ADC-map at the same time. Ideally the morphologic and the functional images should be linked at the same table position. With this described display mode, in many cases a final diagnosis of the primary tumour can already be made according to PI-RADS™ v2. In addition, DCE-MRI is displayed in a continuous stack of images at different time points and table positions. For exact morphologic localisation of suspicious lymph nodes and level of the prostatic lesion isotropic voxel 3D T2w sequence can be reformatted at user's preferences [14]. Additionally fused images of morphology and functional maps may be helpful for exact localisation of findings. A standard layout of our prostate image display is shown in Fig. 2.

### Image interpretation

**Fig. 3:**
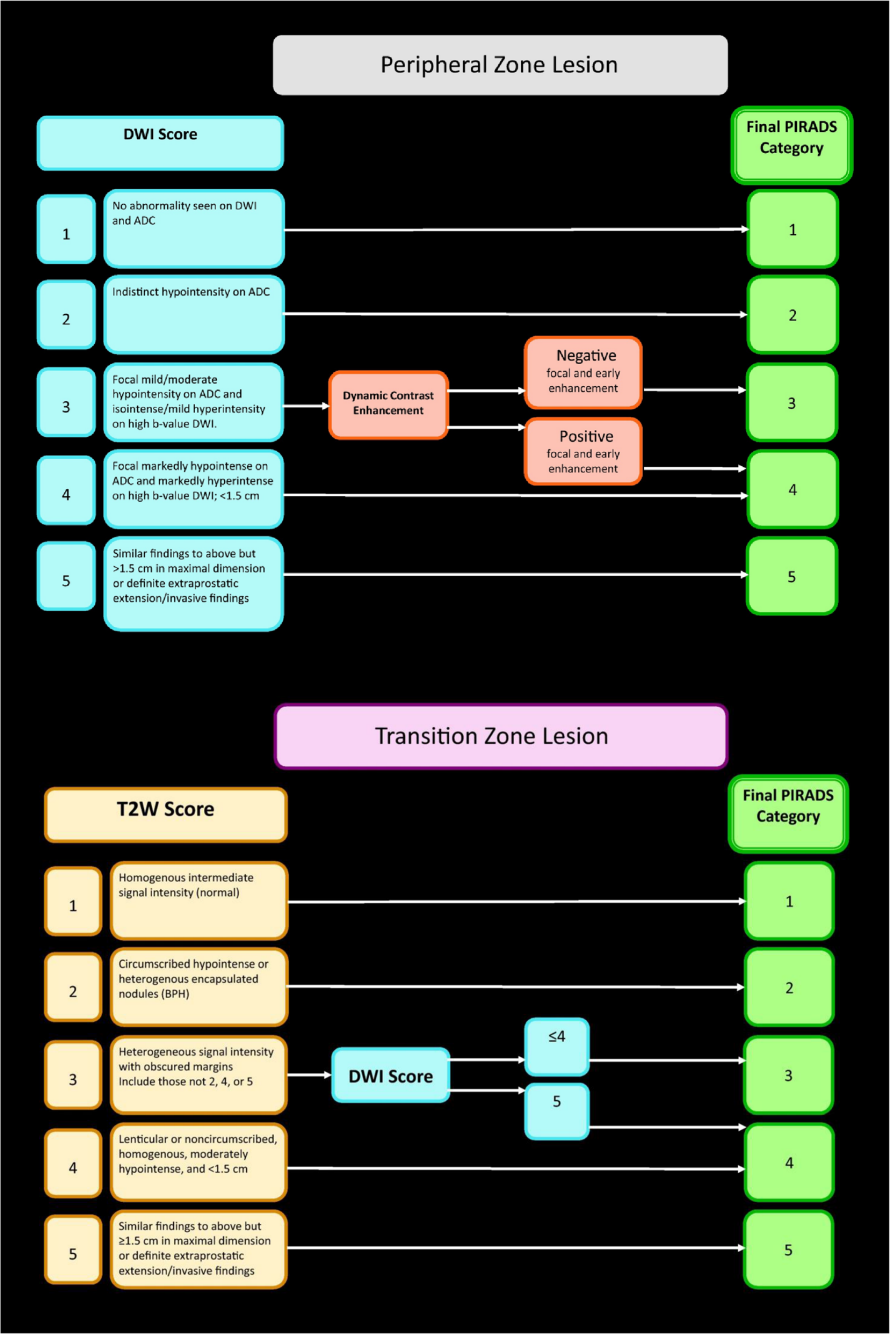
The predominant sequence is DWI in the peripheral zone and T2w sequence in the transition zone. In case of PI-RADS assessment category 3 in the peripheral zone DCE-MRI determines the final PI-RADS assessment category and in PI-RADS assessment category 3 in the transition zone DWI determines the final PI-RADS assessment category. When image quality is compromised and DCE-MRI is inadequate for the assessment of the peripheral zone in PI-RADS assessment category 3 the PI-RADS score is defined by DWI alone. If DWI is not adequate PI-RADS™ v2 guidelines advice use of T2w sequence for both, the peripheral zone and for the transition zone. If both DWI and DCE-MRI are inadequate or not available, assessment should be limited to staging for determination of EPE [11]

Image analysis is based on DWI, DCE-MRI and on T2w sequences. Pattern recognition on these sequences is primarily defined by the criteria of the PI-RADS™ v2 guidelines (Fig. 3) and findings should be described according to the lexicon in appendix III of PI-RADS™ v2 guidelines. Before starting image interpretation, the quality of the images has to be assured. Artefacts like metal implants (e.g. hip prosthesis), air in the rectum and patient movements can compromise the diagnostic value of images. When DWI or DCE-MRI are inadequate, PI-RADS™ v2 guidelines advise to use substitute sequences as described in caption of Fig. 3.

The prostate should be analysed for focal lesions in the prostate and seminal vesicles. Peripheral zone and transition zone are assessed separately as depicted in Fig. 3. Due to the fact that 70-75 % of prostate cancers arise in the peripheral zone, we recommend to start viewing functional images first. In the peripheral zone the dominant sequence is DWI. Suspicious lesions typically have a hyperintense signal intensity on high b-value DW images and a hypointense signal intensity on the corresponding ADC maps. In case a lesion on DWI reveals PI-RADS assessment category 3, the lesion is further assessed on DCE-MRI as positive or negative. DCE-MRI is evaluated as positive, when visual assessment, by either manually scrolling or using cine mode, reveals a focal, earlier or a contemporaneous enhancement to adjacent normal prostatic tissue, and usually corresponds to a suspicious finding on T2w and/or DWI. When such positive findings are detected in a lesion corresponding PI-RADS assessment category 3 on DWI in the peripheral zone, the PI-RADS assessment category is uprated from 3 to 4. Otherwise if DCE-MRI is negative for a lesion in the peripheral zone with PI-RADS assessment category 3 the lesion remains 3.

In the transition zone T2w is the dominant sequence. Suspicious lesions have a heterogeneous signal intensity with obscured margins, lenticular or non-circumscribed homogeneous moderately hypointense signal intensity on T2w images. In case a lesion on T2w imaging reveals PI-RADS assessment category 3, the lesion is further assessed on DWI. When DWI shows markedly hyperintense signal intensity on high b‐value DW images and hypointense signal intensity on the corresponding ADC map of the lesion and the greatest dimension is ≥ 1.5 cm, the lesion is uprated to PI-RADS assessment category 4. All lesions showing EPE and/or invasion of the seminal vesicle correspond to PI-RADS assessment category 5. EPE is defined as a lesion extending to an area outside of the prostate or bulging the capsule of the prostate. Invasion of the seminal vesicle is defined as tumour extension into the seminal vesicle. Thereafter, the entire pelvis should be analysed for lymph node involvement, bone metastases and other findings. Especially DWI sequences of the entire pelvis in combination with meticulous analysis of 3D T2w sequences help to detect lymph node metastases [14]. Pitfalls in prostate cancer imaging can be categorised as previously described [15, 16].

- Normal anatomic structures that may be mistaken for tumour:
  Peripheral zone lesions can be mimicked by normal central zone or periprostatic venous plexus or neurovascular bundle. Bilateral T2w image hypointensities at the base and/or median posterior T2w image hypointense area in the middle third of the gland can mimic peripheral zone lesions. Asymmetric thickening of the surgical capsule can be difficult to distinguish from a focal lesion.
- Noncancerous abnormalities that can mimic tumour:
  Postbiopsy haemorrhage is a pitfall, which can be avoided when PI-RADS™ v2 guidelines are applied correctly and MRI is performed more than 6 weeks after biopsy. The differentiation between stromal benign prostatic hyperplasia and a transition zone tumour can be very difficult. Protrusion of a benign prostatic hyperplasia (BPH) nodule can mimic a peripheral zone lesion. Acute and chronic prostatitis, postinflammatory scars and atrophy mimicking tumour is less difficult to distinguish, due to characteristic patterns on T2w sequences. Without adequate clinical information, granulomatous prostatitis is another mimicker of a tumour and can not be differentiated based on imaging alone.
- Technical challenges related to DWI:
  Anatomic distortion of high b-value DW images can lead to obscuration of focal lesions. Lack of suppression of benign prostate tissue on standard high b-value DW images is another pitfall, which leads to false positive findings. On the other side, suboptimal windowing of ADC maps can yield false negative results.

Experience, meticulous image analysis, including morphological and functional images at the same time are factors, which can reduce false positive results.

### Formulating reports

Reporting at our institution is performed in a structured way on a free text basis. All patients undergoing MRI-TRUS fusion guided biopsy are reported additionally by one senior radiologist specialised in genitourinary radiology on a template scheme map for biopsy planning. This scheme map is further demonstrated to the biopsy performing urologist. According to PI-RADS™ v2 appendix II report templates are under construction. We are looking forward to integrating report templates in computer-aided evaluation soon. Software for computer-aided structured reporting is already on the market for PI-RADS™ v1 and will soon be available for PI-RADS™ v2.

A prostate MRI report should be structured with the following italic subheadings:

#### Clinical notes

According to PI-RADS™ v2 the following information should be available at the time of the exam: PSA and PSA history; information on previous biopsies and corresponding Gleason score; previous surgery and other relevant clinical history of the prostate.

#### Technical details

In this section patient preparation, field strength of the MR scanner, amount and name of contrast medium are mentioned. As the prostate MRI protocol for PI-RADS™ v2 reporting is standardised no further technical details are mentioned. If quality of DWI or DCE-MRI is not adequate, causes of problems should be mentioned in this section and, if possible, performed solutions should be reported in order to perform eventual follow-up studies of the same patient with identical sequence alterations.

#### Findings

In our institution reports start with the prostate volume and an overall impression of the prostatic gland. The prostate gland volume is calculated using the formula for a conventional prolate ellipse: (maximum anterior posterior diameter) x (maximum transverse diameter) × (maximum longitudinal diameter) x 0.52. Findings are described according to PI-RADS™ v2 separately for peripheral zone and transition zone. Lesions are characterised in size, localisation, signal intensity, homo-/heterogeneity, border configuration, possible EPE and possible infiltration of the neurovascular bundle and seminal vesicles as well as infiltration of adjacent organs are reported in this section. Up to four findings based on PI-RADS™ v2 assessment category of 3, 4 or 5 are described and the index (dominant) lesion is identified. Presence or absence of possible lymph node metastases in the entire pelvis are described and exact localisation, number and size are mentioned. Additionally, if present, bone metastases are described. Finally, additional findings in the scanned volume are mentioned.

Key findings are especially described with exact localisation according to PI-RADS™ v2 Appendix III sector map.

#### Conclusion

For each lesion of category 3, 4 and 5 the likelihood for presence or absence of clinically significant prostate cancer is depicted and the exact localisation is mentioned. The corresponding PI-RADS™ v2 score is specified. Presence or absence of extraprostatic extension, lymph node involvement and bone metastases is always explicitly mentioned.

**Fig. 4:**
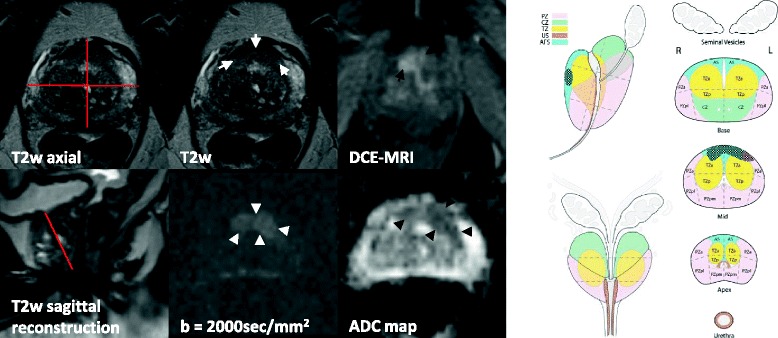
*Clinical Notes:* Actual PSA: 27.5 ng/ml; PSA last year: 31.4 ng/ml. One year previous prostate biopsy revealed no cancer. *Technical Details:*  Standard prostate MRI protocol, 3 T MRI scanner, 16 ml Multihance. *Findings:* Enlarged prostate of circa 43 ml (44 mm × 51 mm × 37 mm × 0.52). Peripheral zone: Midlevel on T2w image depicts a focal lesion of 20 mm maximal extension (PI-RADS 5) (white arrows). The lesion shows a high signal intensity in the anterior fibromuscular stroma and in the left peripheral anterior zone on the high b-value DW image (white arrowheads) with corresponding low signal intensity on the ADC map (black arrowheads) (ADC ~580 × 10^−6^mm^2^/s) (PI-RADS 5). DCE-MRI is rated positive, showing a focal enhancement, earlier than adjacent prostate tissue (black arrows). Transition zone: Circumscribed hypointense encapsulated nodules (BPH) (PI-RADS 2). No suspicious locoregional or pelvic lymph nodes. No suspicious bone lesions. No additional findings. *Conclusion:* Clinically significant prostate cancer is highly likely to be present in the anterior fibromuscular stroma and in the left peripheral anterior zone in the midlevel with greatest dimension ≥ 1.5 cm corresponding to PI-RADS 5. No suspicious lymph nodes or bone lesions. *Histology:* MRI-TRUS fusion guided biopsy revealed Gleason score 4 + 3 = 7

**Fig. 5:**
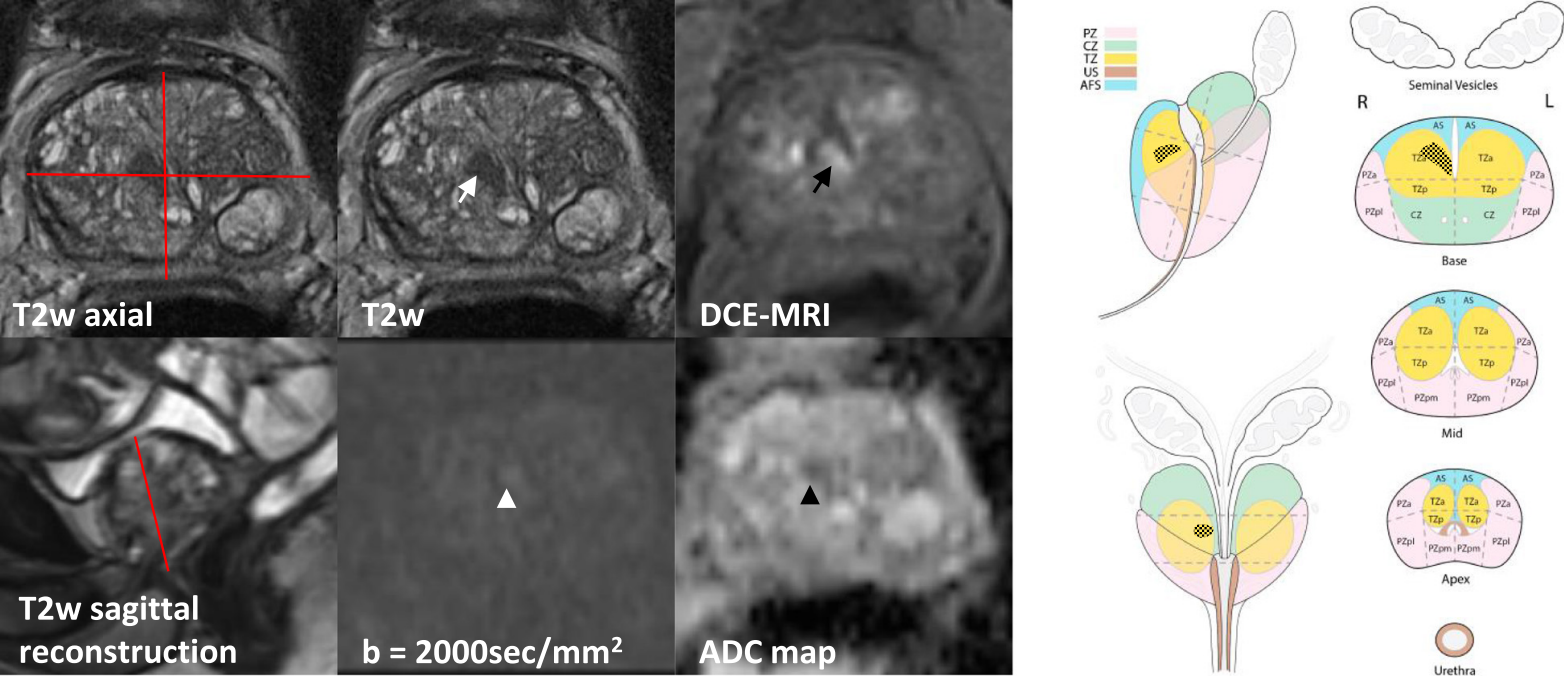
*Clinical Notes:* Known prostate cancer Gleason 6 under active surveillance diagnosed 1 year ago. PSA 5.4 ng/ml. Under α_1_-adrenergic receptor therapy for BPH. *Technical Details:* Standard prostate MRI protocol, 3 T MRI scanner, 13 ml Multihance. *Findings:* Enlarged prostate of circa 48 ml (58 mm × 38 mm × 42 mm × 0.52). Peripheral zone: On high b-value DW images and ADC map normal peripheral zone with indistinct hypointensities on the ADC map (PI-RADS 2). Transition zone: On T2w image lesion of 9 mm maximal extension (white arrow) at the mid-level in the anterior half on the right with heterogeneous, indistinct triangular, signal intensity with obscured margins with mild hypointense signal (PI-RADS 3). The lesion is showing mildly hyperintense signal on high b-value DW image (white arrowhead) and moderate signal intensity on ADC map (black arrowhead) (ADC ~850 × 10^−6^mm^2^/s) (PI-RADS 3). DCE-MRI is rated positive, showing a focal enhancement, earlier than adjacent prostate tissue (black arrow). Multiple circumscribed hypointense encapsulated nodules (BPH) (PI-RADS 2). No suspicious locoregional or pelvic lymph nodes. No suspicious bone lesions. No additional findings. *Conclusion:* The presence of clinically significant prostate cancer is equivocal in the transition zone showing a lesion in the right anterior mid-level corresponding to PI-RADS 3. No suspicious lymph nodes or bone lesions. *Histology: *MRI-TRUS fusion guided biopsy revealed Gleason score 3 + 3 = 6

Two cases and corresponding scheme maps and reports are shown in Figs. 4 and 5.

## Conclusion

Review and reporting of prostate MRI adheres strictly to PI-RADS™ v2 guidelines. Prostate cancer imaging is based on mpMRI, consisting of T2w sequences, DWI and DCE-MRI. Findings on mpMRI are assessed according to the PI-RADS™ v2 5-point category scale. Up to four suspicious lesions of category 3, 4 and 5 of PI-RADS™ v2 are identified and for each lesion the corresponding likelihood (probability) of clinically significant prostate cancer is specified.

Prostate MRI in our institution is performed before MRI-TRUS fusion guided biopsy. In this setting prostate MRI is used for detection and/or staging at the same time. One might even speculate that, when image acquisition gets faster and the cost of examination falls, MRI might be even be used for prostate cancer screening in the future.
